# Characterization of Osterix Protein Stability and Physiological Role in Osteoblast Differentiation

**DOI:** 10.1371/journal.pone.0056451

**Published:** 2013-02-15

**Authors:** Yanyan Peng, Kaikai Shi, Lintao Wang, Jianlei Lu, Hongwei Li, Shiyang Pan, Changyan Ma

**Affiliations:** 1 Department of Developmental Genetics, Nanjing Medical University, Nanjing, People’s Republic of China; 2 Department of Biochemistry and Molecular Biology, Nanjing Medical University, Nanjing, People’s Republic of China; 3 Department of Oral and Maxillofacial Surgery, Affiliated Hospital of Stomatology, Nanjing Medical University, Nanjing, People’s Republic of China; 4 Department of Laboratory Medicine, the First Affiliated Hospital of Nanjing Medical University, Nanjing, People’s Republic of China; 5 National Key Clinical Department of Laboratory Medicine, Nanjing, People’s Republic of China; 6 State Key Laboratory of Reproductive Medicine, Nanjing Medical University, Nanjing, People’s Republic of China; University of Kansas Medical Center, United States of America

## Abstract

Osterix (Osx/SP7) is a C2H2 zinc finger-containing transcription factor of the SP gene family. *Osx* knockout mice indicate that the gene plays an essential role in osteoblast differentiation and bone formation. However, the mechanisms involved in the regulation of Osx are still poorly understood. Here, we report a novel post-translational mechanism for the regulation of Osx in mammalian cells. We found that the stability of endogenous and exogenous Osx reduced after cycloheximide treatment. In cells treated with the proteasome inhibitors MG-132 or lactacystin, both endogenous and exogenous Osx protein expression increased in a time-dependent manner. Co-immunoprecipitation (Co-IP) assays showed that both endogenous and exogenous Osx were ubiquitinated. Six lysine residues of Osx were identified as candidate ubiquitination sites by construction of point mutant plasmids and luciferase reporter assays. Furthermore, we confirmed that K58 and K230 are the ubiquitination sites of Osx by Co-IP assays and protein stability assays. Moreover, the Osx K58R and K230R mutations promoted the expression of osteoblast differentiation markers (alkaline phosphatase, collagen I and osteocalcin) and enhanced osteogenic differentiation in C2C12 cells. Taken together, our data indicate that Osx is an unstable protein, and that the ubiquitin-proteasome pathway is involved in the regulation of Osx and thereby regulates osteoblast differentiation.

## Introduction

Bone is continuously destroyed and reformed to maintain constant bone volume and calcium homeostasis in vertebrates throughout their lives [1]. Osteoblasts and osteoclasts are specialized cells responsible for bone formation and resorption, respectively [1]–[3]. The proper balance between osteoblasts and osteoclasts is essential for maintaining bone function. The activation of osteoblasts is regulated at the level of differentiation from mesenchymal stem cells, which is controlled by various transcription factors and signaling proteins, including the Wnt signaling pathway, Indian Hedgehog, Runt-related transcription factor 2 (Runx2) and Osterix (Osx/Sp7) [4].

Osx, a zinc finger-containing transcription factor, plays a critical role in osteoblast differentiation and bone formation, as no bone formation occurs in *Osx* knockout mice [5]–[8]. It has been reported that Osx is required for bone morphogenetic protein 2 (BMP-2) and Runx2-induced osteoblast differentiation and bone growth in both postnatal and adult mice [9], [10]. As a gene downstream of Runx2, *Osx* is specifically expressed in osteoblasts and expressed at low levels in prehypertrophic chondrocytes [5]. The pattern of *Osx* expression in mice indicates that the *Osx* transcript is expressed as early as the commitment time for mesenchymal cells to enter the osteoblast lineage, and expression of *Osx* becomes stronger as osteoblast differentiation occurs. At present, the regulation of Osx is not fully understood, and knowledge of the post-translational regulation of Osx is especially lacking.

Post-translational modifications are important for regulating the function, localization and turn-over of cellular protein impacting on cellular morphology, activity and interactions within multi-cellular organisms [11]. Ubiquitination is one example of post-translational modifications, and regulates a broad range of cellular processes including metabolic adaptations, cell cycle progression, cell differentiation and also signaling and gene regulation. Ubiquitination of protein substrates proceeds via a step-wise process involving three enzymes: ubiquitin-activating enzyme (E1), ubiquitin-conjugating enzyme (E2), and ubiquitin ligase (E3). Poly-ubiquitylated proteins are then targeted to the 26S proteasome for degradation [12]–[14]. Ubiquitination, along with other post-translational protein modifications such as phosphorylation, hydroxylation and acetylation, is a highly regulated process for the execution of which a multitude of regulators exist [11].

To investigate whether ubiquitination modifications are involved in the regulation of Osx, and whether turn-over of Osx by the ubiquitin-proteasome system (UPS) plays a role in osteoblast differentiation, we examined the stability of Osx and investigated the influence of the UPS on the function of Osx. We found that Osx is an unstable protein. In addition, we report here for the first time that the UPS is involved in the regulation of Osx. Moreover, we identified that K58 and K230 are the ubiquitination sites in Osx, and demonstrated that ubiquitination of Osx plays an important role in osteoblast differentiation. These data suggest that the ubiquitin-proteasome pathway (UPP) plays a role in osteoblast differentiation and bone formation via regulating the expression of Osx. Our results shed some light on the mechanisms of Osx regulation and further elucidation of the regulation of bone metabolism.

## Materials and Methods

### Cell Culture

Saos-2 cells were maintained in McCoy’s 5A medium supplemented with 15% fetal bovine serum (FBS). MG-63 cells were maintained in minimum Eagle’s medium (MEM) supplemented with 10% FBS. HEK 293T, A549 and SW1990 cells were cultured in high glucose Dulbecco’s modified Eagle’s medium (DMEM) supplemented with 10% FBS. C2C12 cells were maintained in DMEM supplemented with 15% FBS. All cells were purchased from the ATCC (Manassas, VA, USA) and cultured in the presence of 100 units/ml penicillin and 100 µg/ml streptomycin at 37°C in 5% CO_2_.

### Reagents and Antibodies

Cycloheximide (CHX), MG-132 and lactacystin were purchased from Sigma Chemical Company (St Louis, MO, USA). Recombinant human BMP-2 (rhBMP-2) was obtained from R&D Systems (Minneapolis, MN, USA). Anti-Osx antibody was obtained from Abcam (Cambridge, MA, USA). Anti-Flag antibody and anti-HA antibody were obtained from Sigma. Anti-Ubiquitin (Ub) antibody was obtained from Santa Cruz (Santa Cruz, CA, USA). Anti-β-actin antibody, anti-rabbit and anti-mouse IgG antibody were obtained from Bioworld Technology (Minneapolis, MN, USA).

### Plasmid Construction

The human Osx expression construct (pcDNA3.1-Flag-hOsx) was generated by subcloning polymerase chain reaction (PCR)-amplified full-length *Osx* cDNA (from Saos-2 cell cDNA) into the *Bam*HI and *Xho*I sites of a pcDNA3.1 expression vector containing Flag-tag at the N-terminal portion. The sequence of *Osx* cDNA was confirmed by DNA sequence analysis.

Lys (K) to Arg (R) mutants of Osx were generated using the Quickchange site-directed mutagenesis kit (Stratagene, La Jolla, CA, USA). Briefly, the *Osx* cDNA template was denatured at 95°C. The mutagenic primers containing the desired mutation were annealed at 60°C and the primers extended using *Pfu*Ultra DNA polymerase at 68°C. The parental DNA was digested with the restriction enzyme *Dpn*I. The pure, mutated DNA was transformed into competent cells and harvested. The mutated fragment was confirmed by sequencing.

A luciferase reporter construct containing the human osteocalcin (Ocn) promoter (Ocn-Luc) was prepared using the pGL3-basic vector (Promega, Mannheim, Germany). The human *Ocn* promoter (391 bp) containing the Osx binding sites was PCR-amplified from human genomic DNA with appropriate primers using standard PCR conditions. The PCR products were resolved on an agarose gel, and the correctly sized fragments were recovered using a DNA Gel extraction kit (Millipore, Bedford, MA, USA). The purified PCR products and pGL3-basic vector were digested with the appropriate restriction enzymes and used to construct Ocn-Luc recombinant vectors. The *Ocn* promoter fragment was confirmed by sequencing.

### Western Blot Assay

Cells were lysed with RIPA buffer (50 mM Tris-HCl, pH 7.5, 150 mM NaCl, 1% NP-40, 0.5% sodium deoxycholate and 0.1% SDS) containing protease inhibitors (Roche Diagnostics, Indianapolis, IN, USA). Total protein (20 µg) was boiled for 5 min in 1× loading buffer, chilled on ice, and then separated on 8% SDS-polyacrylamide gels. Following transfer onto PVDF membranes (Millipore), nonspecific protein interactions were blocked by incubation in 5% non-fat dry milk in TST-buffer (50 mM Tris-HCl, 150 mM NaCl, 0.05% Tween-20 pH 7.6) at room temperature (RT) for 1 h. Membranes were then incubated overnight at 4°C with primary antibody in fresh blocking buffer. Unbound antibody was removed by three 10 min washes in TST buffer. Membranes were then incubated with horseradish peroxide-conjugated secondary antibody for 1 h at RT, followed by three 10 min washes with TST buffer. The blots were developed using an enhanced chemiluminescence system (Pierce, Rockford, IL, USA). Prestained markers were used as molecular weight standards.

### Co-immunoprecipitation (Co-IP) Assay

For Co-IP of endogenous proteins, Saos-2 cells were washed twice with ice-cold phosphate-buffered saline (PBS), lysed with RIPA buffer plus protease inhibitors and MG-132, and 500 µl aliquots of the cell lysates were incubated overnight at 4°C with anti-Osx or anti-Ub antibody. To capture the immunocomplexes, 30 µl of protein-G agarose beads (Millipore) were added and incubated at 4°C for 4 h. The agarose slurry was collected by centrifugation and the supernatants were discarded. The pellets were washed three times with RIPA buffer, then resuspended in SDS gel-loading buffer and analyzed by SDS-PAGE followed by Western blot analysis using anti-Ub or anti-Osxantibody.

For Co-IP of overexpressed proteins, lysates from HEK 293T cells co-transfected with pcDNA3.1-Flag-hOsx and HA-tagged ubiquitin expression plasmids (a generous gift of Dr. X Cao, Department of Pathology, University of Alabama at Birmingham, AL, USA) were used. The Co-IP was performed as above using anti-Flag antibody to precipitate the immunocomplexes and the anti-HA antibody to perform Western blot analysis.

### Luciferase Reporter Assay

HEK 293T cells were co-transfected with the Ocn-Luc reporter, Renilla luciferase reporter vector, the WT Flag-hOsx plasmid or Lys-to-Arg mutants. After 36 h of transfection, luciferase activity was measured using the Dual Luciferase Reporter System (Promega) according to the manufacturer’s protocol. Relative luciferase activity was calculated by dividing the firefly luciferase activity by the Renilla luciferase activity. Data are representative of three independent experiments performed in triplicate.

### RNA Isolation

Total RNA was isolated from C2C12 cells transiently transfected with pcDNA3.1-Flag-hOsx (WT), K58R, K230R mutants or empty pcDNA3.1 vector using TRIzol reagent (Invitrogen, Carlsbad, CA, USA) according to the manufacturer’s protocol. The RNA was digested with RQ1 RNase-Free DNase (Promega) for 30 min to remove genomic contaminants. Subsequently, DNase was removed with the RNeasy Mini Kit (Qiagen, Hilden, Germany) and absence of residual genomic DNA was confirmed by PCR analysis. Visualization of the ribosomal bands on a 1% TAE agarose gel was used to assess RNA integrity.

### Real-time RT-PCR

Briefly, cDNA was synthesized from 1 µg of total RNA using the SuperScript synthesis system (Applied Biosystems), according to the manufacturer’s instructions. Real-time PCR was carried out using the ABI 7500 Real-time PCR system (Applied Biosystems). The reactions (20 µl) contained cDNA, forward and reverse primers and SYBR GREEN PCR Master Mix (Applied Biosystems). The amplification conditions were 50°C for 2 min, 95°C for 10 min, followed by 40 cycles of denaturation at 95°C for 15 s and annealing and extension at 60°C for 1 min. The following primers were used: *ALP*, Forward: 5′-TGACCTTCTCTCCTCCATCC-3′, Reverse: 5′-CTTCCTG GGAGTCTCATCCT-3′; *Col I*, Forward: 5′-GCAACAGTCGCTTCACCTACA-3′, Reverse: 5′-CAATGTCCAAGG GAGCCACAT-3′; *Ocn*, Forward: 5′-TGCTTGTG ACGAGCTATCAG-3′, Reverse: 5′-GAGGACAGGGAGGATCAAGT-3′; *Osx*, Forward: 5′-AGCGACCACTTGAGCAAACAT-3′, Reverse: 5′-GCGGCTGATTGG CTTCTTCT-3′; *β-actin*, Forward: 5′-AGATGTGGATCAGCAAGCAG-3′, Reverse: 5′-GCGCAAGTTAGGTTTTGTCA-3′.

### Alkaline Phosphatase (ALP) Staining

C2C12 cells seeded in 24-well plates were transfected with pcDNA3.1-Flag-hOsx (WT), K58R, K230R mutants or empty pcDNA3.1 vector using Lipofectamine 2000 (Invitrogen, Carlsbad, CA, USA). At 24 h after transfection, the cells were treated with BMP-2 (100 ng/ml) for 5–7 days. Before staining, the transfected C2C12 cells were fixed in 10% paraformaldehyde for 10 min at RT. After washing with PBS, the cells were stained with 300 µg/ml of BCIP/NBT solution for 20 min at RT; alkaline phosphatase-positive cells stained blue/purple.

### Mineralization Assay: Alizarin Red Staining (ARS)

C2C12 cells seeded in 24-well plates were transfected with pcDNA3.1-Flag-hOsx (WT), K58R, K230R mutants or empty pcDNA3.1 vector using Lipofectamine 2000. At 24 h after transfection, the cells were treated with BMP-2 (100 ng/ml) for 10–12 days. After the cells were fixed in 5% paraformaldehyde for 10 min, the cells were stained with 2% ARS (pH 7.2) for 15 min and then washed twice with PBS. The orange and red areas were scored as calcium nodules.

### Statistical Analysis

Results are expressed as mean ± SD. The Student’s *t*-test and analysis of variance (ANOVA) were used to assess differences; *P*<0.05 was considered significant.

## Results

### Osx is Differentially Expressed in Various Cell Lines

**Figure 1:**
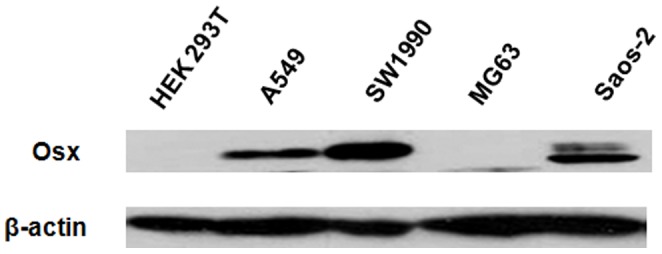
Screening for suitable cell models. Whole cell extracts of HEK 293T, A549, SW1990, MG-63 and Saos-2 cells were isolated and analyzed by Western blotting with an anti-Osx antibody. β-actin served as a loading control.

To select a suitable cell model, we screened the expression of Osx in various cell lines including human embryonic kidney HEK 293T cells, human lung carcinoma A549 cells, human pancreatic adenocarcinoma SW1990 cells, human osteosarcoma MG-63 cells and Saos-2 cells by Western blot analysis. The expression of Osx was relatively high in Saos-2 cells, which are usually used as a model of osteoblast cells, as well as in A549 and SW1990 cells. However, the expression of Osx was barely detectable in HEK 293T and MG-63 cells (Figure 1). Therefore, Saos-2 and HEK 293T cells were selected as the cell models for modification of endogenous and exogenous Osx expression, respectively.

### Osx is a Short-lived Protein

**Figure 2:**
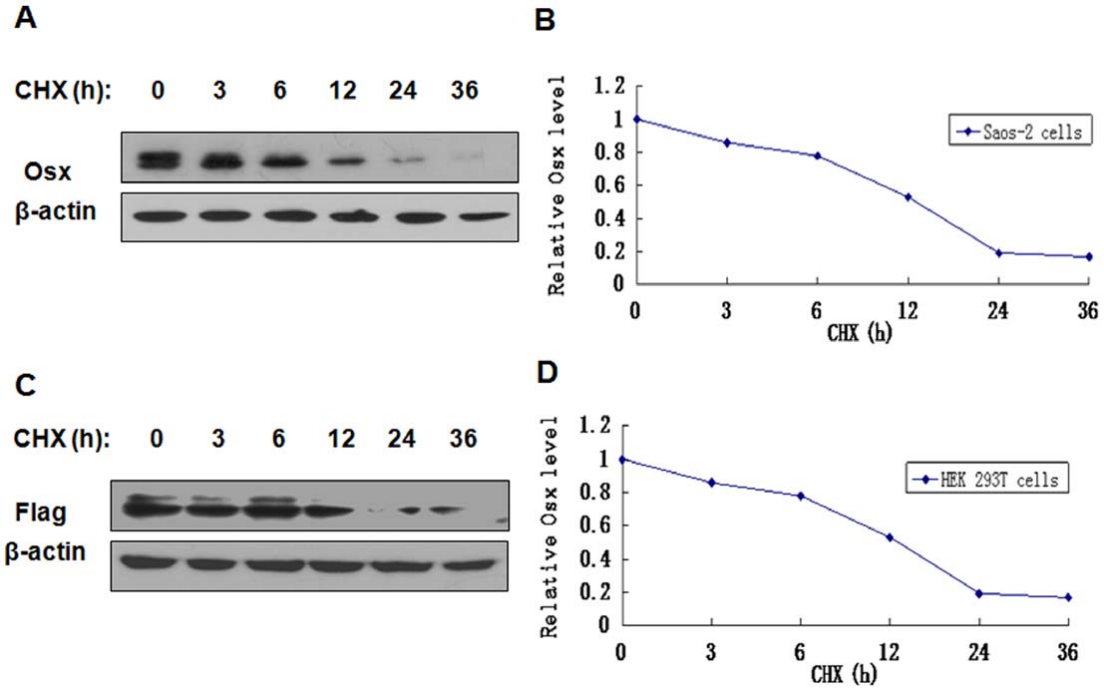
Osx is a short-lived protein. A. Saos-2 cells were treated with CHX (80 µM) for various periods of time and endogenous Osx protein expression was detected by Western blot analysis with an anti-Osx antibody. B. The expression levels of Osx in panel A were determined by densitometry. The levels of Osx protein in CHX-untreated cells (0 h) were set to 100%. C. HEK 293T cells were transfected with Flag-hOsx plasmids and then treated with CHX (80 µM) for various periods of time. Exogenous Osx protein was detected by Western blot analysis with an anti-Flag antibody. β-actin served as a loading control. D. The expression levels of Osx in panel B were determined by densitometry. The levels of Osx protein in CHX-untreated cells (0 h) were set to 100%. Results are shown for one of three independent experiments.

To examine the stability of Osx protein, Saos-2 cells were treated with the protein synthesis inhibitor CHX, and then Osx expression was measured by Western blot analysis. As shown in Figure 2A and 2B, the levels of endogenous Osx protein expression decreased after 80 µM CHX treatment in a time-dependent manner. The half-life of endogenous Osx was approximately 12 h. Additionally, HEK 293T cells transfected with Flag-hOsx plasmids were treated with 80 µM of CHX for the indicated time points. Exogenous Osx protein was also unstable, with a half-life of approximately 12 h (Figures 2C and 2D).

### Inhibition of Proteasomal Activity Blocks the Degradation of Osx

**Figure 3:**
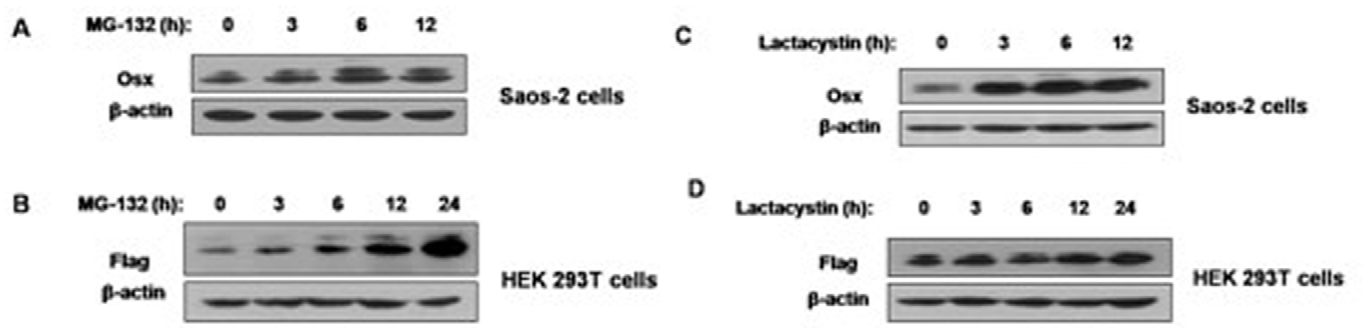
Both endogenous and exogenous Osx are increased by proteasome inhibitor treatment. A. Saos-2 cells were treated with MG-132 (20 µM) for various periods of time. Endogenous Osx protein was detected by Western blot analysis with an anti-Osx antibody. B. HEK 293T cells were transfected with Flag-hOsx plasmids and then treated with MG-132 (20 µM) for various periods of time. Exogenous Osx protein was detected by Western blot analysis with an anti-Flag antibody. C. Saos-2 cells were treated with lactacystin (10 µM) for various periods of time. Endogenous Osx protein was detected by Western blot analysis with an anti-Osx antibody. D. HEK 293T cells were transfected with Flag-hOsx plasmids and then treated with lactacystin (10 µM) for various periods of time. Exogenous Osx protein was detected by Western blot analysis with an anti-Flag antibody. β-actin served as a loading control. Results are shown for one of three independent experiments.

To determine the role of the proteasomal pathway in the degradation of Osx, Saos-2 cells, or HEK 293T cells transfected with Flag-hOsx plasmids, were treated with protease inhibitors and the levels of Osx were assessed by Western blot analysis. As shown in Figures 3A and 3B, 20 µM of MG-132 led to a marked increase in both endogenous and exogenous Osx in a time-dependent manner. Moreover, Saos-2 cells, and HEK 293T cells transfected with Flag-hOsx plasmids, were treated with another protease inhibitor lactacystin. Similarly to MG-132 treatment, significant increases in both endogenous and exogenous Osx were observed in a time-dependent manner after treatment with 10 µM of lactacystin (Figures 3C and 3D). These data suggest that the proteasomal pathway may be, at least in part, responsible for the degradation of Osx.

### Osx Protein is Ubiquitinated

**Figure 4:**
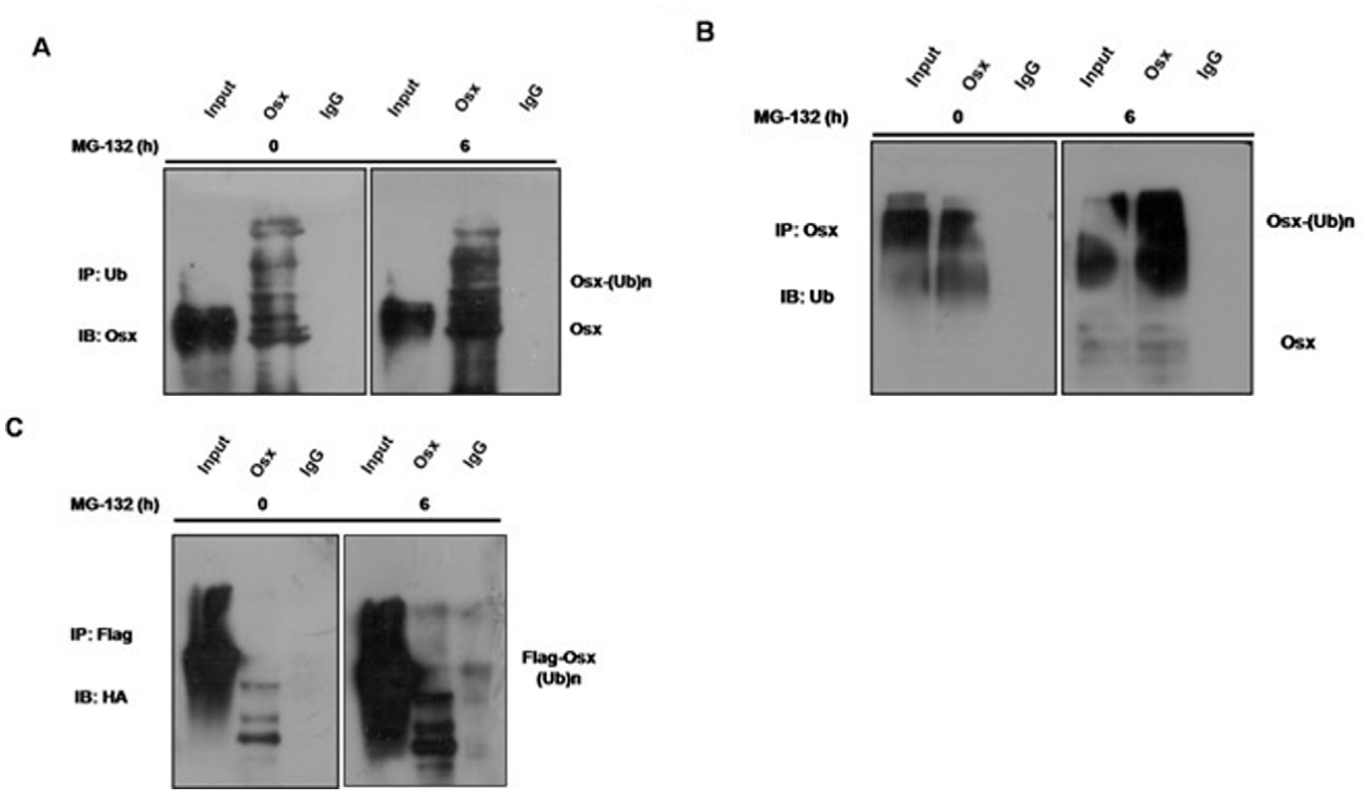
Both endogenous and exogenous Osx are ubiquitinated. A. Saos-2 cells treated with or without MG-132 (20 µM) for 6 h were lysed, the cell lysates were immunoprecipitated with an anti-Ub antibody, and then blotted with an anti-Osx antibody. B. Saos-2 cells treated with or without MG-132 (20 µM) for 6 h were lysed, the cell lysates were immunoprecipitated with an anti-Osx antibody, and then blotted with an anti-Ub antibody. C. HEK 293T cells were transiently co-transfected with Flag-tagged hOsx and HA-tagged ubiquitin plasmids, and then treated with or without MG-132 (20 µM) for 6 h. The cell lysates were immunoprecipitated with an anti-Flag antibody, and then blotted with an anti-HA antibody. Results are shown for one of three independent experiments.

Proteasomes are multi-subunit complexes that selectively degrade ubiquitinated proteins. To determine whether Osx protein is ubiquitinated prior to its degradation by the proteasome, immunoprecipitation assays were performed in both Saos-2 cells and Osx-transfected HEK 293T cells. Saos-2 cells were lysed after treatment with or without 20 µM of MG-132 for 6 h, and Osx was immunoprecipitated using an anti-Ub antibody and immunoblotted with an anti-Osx antibody. As shown in Figure 4A, a ladder of mono- and polyubiquitinylated Osx was detected in both treated and untreated Saos-2 cells. However, MG-132 treatment dramatically increased the levels of ubiquitinated Osx. Mono- and polyubiquitinylated Osx were also detected in both treated and untreated Saos-2 cells when immunoprecipitated using an anti-Osx antibody and immunoblotted with an anti-Ub antibody (Figure 4B).

To determine whether exogenous Osx protein is also ubiquitinated, Flag-tagged hOsx and HA-tagged ubiquitin plasmids were co-transfected into HEK 293T cells. At 24 h after transfection, the cells were treated with or without MG-132 for 6 h. The cell lysates were immunoprecipitated using an anti-Flag antibody and blotted with an anti-HA antibody. As shown in Figure 4C, a ladder of mono- and polyubiquitinylated Osx was detected, indicating that exogenous Osx protein was also ubiquitinated.

### Identification of the Ubiquitination Sites in Osx

**Figure 5:**
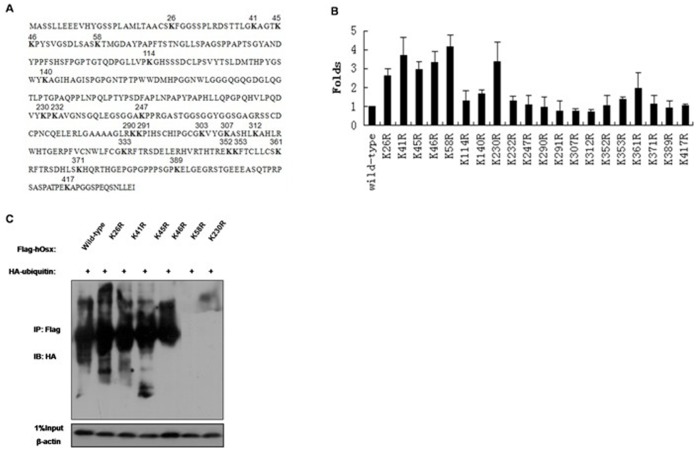
Identification of the ubiquitination sites in Osx. A. Osx primary sequence, with the lysine residues indicated. B. HEK 293T cells were co-transfected with Ocn-Luc reporter, control Renilla luciferase reporter vector, and the WT Flag-hOsx plasmid or Lys-to-Arg mutants. Relative luciferase activity was measured 24 h after transfection and normalized to the Renilla activity. Results were obtained from three independent experiments performed in triplicate. Data are expressed as mean ± SD. C. HEK 293T cells were co-transfected with HA-Ub expression plasmids and the WT Flag-hOsx plasmid or K26R, K41R, K45R, K46R, K58R and K230R mutants. Cell lysates were immunoprecipitated using an anti-Flag antibody and blotted with an anti-HA antibody. Results are shown for one of three independent experiments.

To address the effects of polyubiquitination of Osx on its activity, it is critical to map the Ub Lys acceptor site(s) of Osx, and characterize the functional effects of eliminating these site(s) on Osx activity. We systematically replaced each of the Osx Lys residues (Figure 5A) with an Arg residue, which would maintain the positive charge but would not serve as an acceptor site for Ub modification. The WT Flag-hOsx plasmid or mutants with different Lys-to-Arg mutations were co-transfected along with Ocn-Luc constructs into the Osx-deficient HEK 293T cells. As shown in Figure 5B, overexpression of the K26R, K41R, K45R, K46R, K58R and K230R mutants increased Ocn-dependent luciferase reporter gene expression by more than 2.5-fold, compared with WT Osx, indicating that the Lys-to-Arg mutations at these sites abolish Osx polyubiquitination and degradation, and result in an increased Osx transactivity. Therefore, one or more of these Lys residues are potential Ub acceptor site(s) which mediate Osx polyubiquitination.

To further map the Ub Lys acceptor site(s) which mediate Osx polyubiquitination, we co-transfected the expression vectors expressing WT Flag-tagged Osx or the six Lys-to-Arg mutants described above, along with a HA-ubiquitin expression vector, into HEK 293T cells. Flag-Osx proteins were immunoprecipitated with an anti-Flag antibody and immunoblotted with an anti-HA antibody. In this assay, we found that only the K58R and K230R mutants displayed dramatically decreased polyubiquitination (Figure 5C), suggesting that these two residues may be the ubiquitination sites of Osx.

### The Osx K58R and K230R Mutant Proteins are More Stable than WT Osx

**Figure 6:**
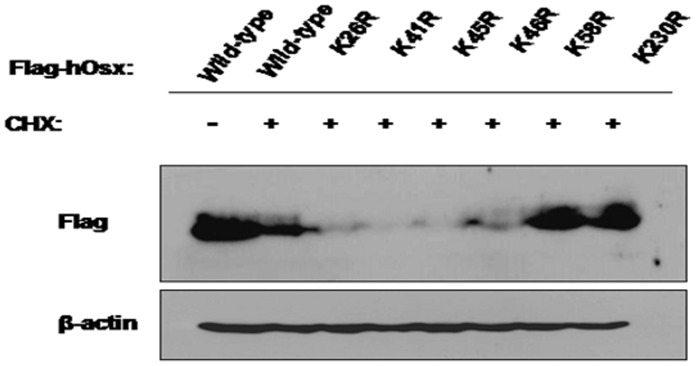
The Osx K58R and K230R mutant proteins are more stable than WT Osx. HEK 293T cells were transiently transfected with WT Flag-hOsx plasmid or the K26R, K41R, K45R, K46R, K58R and K230R mutants, and treated with CHX (80 µM) for 12 h. Cell lysates were assessed by Western blot analysis with an anti-Flag antibody. β-actin served as a loading control. Images are representative of three independent experiments.

To further confirm that K58 and K230 are the ubiquitination sites of Osx, the stability of the six Lys-to-Arg mutants described above was examined. We transfected WT Flag-hOsx plasmid or the six Lys-to-Arg mutants into HEK 293T cells, and 24 h later the cells were treated with CHX (80 µM) for 12 h. The levels of Osx were determined by Western blot analysis with an anti-Flag antibody. We found that both the K58R and K230R mutants were more stable than the other four mutants, K26R, K41R, K45R and K46R (Figure 6). In conjunction with the Co-IP results above, this data demonstrated that K58 and K230 are the ubiquitination sites of Osx.

### The Osx K58R and K230R Mutations Enhance the mRNA Expression Levels of Osteoblast Differentiation Marker Genes in C2C12 Cells

**Figure 7:**
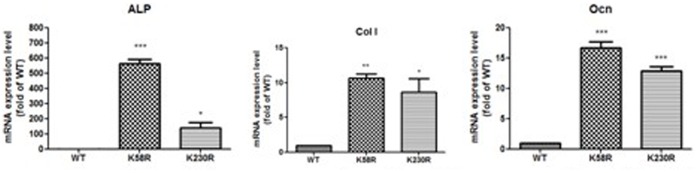
The Osx K58R and K230R mutations enhance the mRNA expression of osteoblast differentiation marker genes. C2C12 cells were transiently transfected with the WT Flag-hOsx plasmid or the K58R and K230R mutants, and 24 h later total RNA was extracted and *ALP*, *Col I* and *Ocn* mRNA were quantified by real-time PCR analysis and normalized to β-actin. Results are mean ± SD of three independent experiments performed in triplicate; *p<0.05, **p<0.01 and ***p<0.001.

Osx regulates the expression of several marker genes of osteoblast differentiation, such as *ALP*, *Col I* and *Ocn*. To examine the effects of Osx ubiquitination on osteoblast differentiation, we transiently transfected the WT Flag-hOsx plasmid or K58R and K230R mutants into C2C12 cells. The mRNA levels of *ALP*, *Col I* and *Ocn* were detected by real-time PCR analysis. The mRNA expression levels of *ALP*, *Col I* and *Ocn* dramatically increased in C2C12 cells transfected with the K58R or K230R mutants, compared to cells transfected with the WT Flag-hOsx plasmid (Figure 7).

### The Osx K58R and K230R Mutations Enhance ALP Activity and Mineralized Matrix Formation in C2C12 Cells

**Figure 8:**
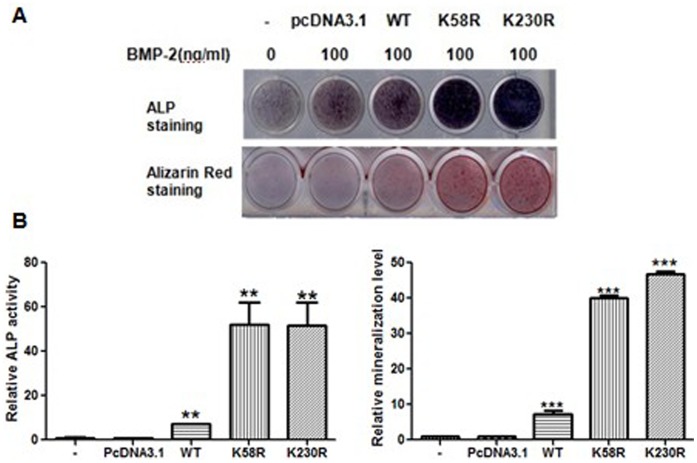
The Osx K58R and K230R mutations enhance ALP activity and mineralized matrix formation. C2C12 cells were transiently transfected with pcDNA3.1, the WT Flag-hOsx plasmid or the K58R and K230R mutants. At 24 h after transfection, the cells were incubated with rhBMP-2 (100 ng/ml). ALP activity was examined by ALP staining 5–7 days later, or mineralization was assessed using Alizarin Red staining 10–12 days later. A. Representative images of three independent experiments are shown. B. Quantification of ALP activity and mineralization shown in A. Results are mean ± SD of three independent experiments; **p<0.01 and ***p<0.001.

We also examined the effects of the Osx K58R and K230R mutants on osteoblastic differentiation using ALP staining and ARS staining assays. We transfected the WT Flag-hOsx plasmid or K58R and K230R mutants into C2C12 cells, and 24 h after transfection the cells were treated with rhBMP-2 (100 ng/ml). ALP staining showed that ALP activity in C2C12 cells transfected with the K58R and K230R mutants was obviously increased compared to cells transfected with the WT Flag-hOsx plasmid (Figures 8A and 8B). ARS staining showed that mineralization in C2C12 cells transfected with the K58R and K230R mutants was markedly enhanced compared to cells transfected with the WT Flag-hOsx plasmid (Figures 8A and 8B). These data suggest that ubiquitination of Osx may play a role in directing the differentiation of C2C12 cells towards the osteoblast lineage.

## Discussion

Osx is a master transcription factor for osteoblast differentiation and bone formation [5]. In this study, we demonstrated that Osx is a short-lived protein, and that the UPS is involved in the regulation of Osx. Furthermore, we identified that the K58 and K230 residues are the ubiquitination sites of Osx. Moreover, we demonstrated that ubiquitination of Osx plays an important role in osteoblast differentiation.

Post-translational modifications of transcription factors such as phosphorylation, acetylation and ubiquitination play a key role in controlling numerous biological events, including cellular energy metabolism, cell-cycle progression and organ development [15]. To determine whether ubiquitination is involved in the regulation of Osx, we firstly examined Osx protein stability using CHX treatment. We found that Osx is an unstable protein with a half-life of 12 h. In eukaryotic cells, there are two mechanisms for protein degradation: the lysosome pathway, a nonspecific process, and the UPP [16]. In higher eukaryotes, the vast majority (80–90%) of intracellular proteins are degraded via the UPP [17], [18]. Ubiquitination-dependent proteasomal degradation is involved in the regulation of numerous cellular processes, including cell cycle progression, apoptosis, DNA repair, the maintenance of cellular quality control, autophagy, the regulation of transcription and receptor-mediated endocytosis [19]–[21]. To determine whether the stability of Osx protein is regulated by the UPP, we examined the changes in Osx protein expression in cells treated with the proteasome inhibitors MG-132 and lactacystin. We found that the stability of Osx markedly increased after MG-132 and lactacystin treatment, suggesting that the UPP is, at least in part, responsible for the degradation of Osx.

In the canonical UPP, substrate proteins are first tagged by multiple ubiquitin molecules and then degraded by the 26S proteasome. The activating enzyme E1 forms a high-energy thioester bond with ubiquitin (Ub), which is then transferred to a reactive cysteine residue of the conjugating enzyme E2. The final transfer of Ub to an ε-amino group of a reactive Lys residue of the substrate protein is brought about by E3, the ubiquitin ligase enzyme [22], [23]. The identification of ubiquitination sites provides the ultimate proof that the putative substrate is indeed ubiquitinated [24]. Therefore, we screened for potential ubiquitination sites in Osx by construction of Lys to Arg mutant plasmids and luciferase reporter assays. We found that the K26R, K41R, K45R, K46R, K58R and K230R mutants induced markedly increased Ocn-dependent luciferase reporter gene expression, indicating that these residues may be ubiquitination sites. To further map the ubiquitination sites, we performed Co-IP assays and measured Osx protein stability. These experiments demonstrated that K58 and K230 are the ubiquitination modification sites in Osx. However, the detailed mechanisms of ubiquitination modifications for Osx need to be investigated further.

Modification of proteins by ubiquitination is a critical regulatory mechanism for various cellular processes [25]. To determine the function of the ubiquitination of Osx in osteoblast differentiation, we transfected the WT Flag-hOsx plasmid or K58R and K230R mutants into pluripotent mesenchymal precursor C2C12 cells, which provide a suitable model system to study the early stage of osteoblast differentiation during bone formation [26]. The mRNA expression levels of osteoblast differentiation markers including *ALP, Col I* and *Ocn* markedly increased in K58R and K230R mutant-transfected cells. Moreover, enhanced ALP activity and increased numbers of mineralized nodules were detected in K58R and K230R mutant-transfected cells, indicating that ubiquitination of Osx plays an important role in osteoblast differentiation. It has been reported that the UPP exerts exquisite control of osteoblast differentiation and bone formation in vitro and in vivo [27]–[29]. Inhibition of the UPP by proteasome inhibitors promotes osteoblast differentiation in vitro and bone formation in vitro and in vivo [28]. Our data suggests that the UPP can control osteoblast differentiation and bone formation through regulation of Osx.

In summary, we show here for the first time that the UPP is involved in the regulation of Osx. Moreover, we identified that the K58 and K230 residues are the ubiquitination sites of Osx, and demonstrated that ubiquitination of Osx plays a key role in osteoblast differentiation. This study reveals new information on the mechanisms of post-translational regulation by which the UPP regulates the osteoblast-specific transcription factor Osx during osteoblast differentiation.
